# The Intra- or Extracellular Redox State Was Not Affected by a High vs. Low Glycemic Response Diet in Mice

**DOI:** 10.1371/journal.pone.0128380

**Published:** 2015-06-01

**Authors:** Amber S. Kleckner, Siu Wong, Barbara E. Corkey

**Affiliations:** Boston University School of Medicine, Boston, Massachusetts, United States of America; University of Cordoba, SPAIN

## Abstract

A low glycemic response (LGR) vs. high glycemic response (HGR) diet helps curtail the development of obesity and diabetes, though the mechanisms are unknown. We hypothesized that consumption of a HGR vs. a LGR diet would lead to a more oxidized circulating redox state and predicted that a HGR diet would increase fat accumulation, reduce insulin sensitivity, and impair metabolic acclimation to a high fat diet in a mouse model. Hence, male C57BL/6 mice consumed a HGR or LGR diet for 16 weeks and a subset of the mice subsequently consumed a high fat diet for 4 weeks. We found that body mass increased at a faster rate for those consuming the HGR diet. Percent body fat was greater and percent lean mass was lesser in the HGR group starting at 12 weeks. However, the groups did not differ in terms of glucose tolerance at week 14 and metabolic parameters (respiratory exchange ratio, heat production, activity) at weeks 4 or 15. Moreover, mice on either diet did not show differences in metabolic acclimation to the high fat leg of the study. At the termination of the study, the groups did not differ in terms of redox pairs (lactate/pyruvate and β-hydroxybutyrate/acetoacetate) or thioredoxin reductase activity in blood. Also, total and oxidized glutathione levels and lipid peroxidation were similar in blood and liver. Correlations between baseline measures, longitudinal parameters, environmental conditions, and terminal metrics revealed that individual mice have innate propensities to metabolic regulation that may be difficult to perturb with diet alone; for example, starting mass correlated negatively with energy expenditure 4 weeks into the study and total hepatic glutathione at the end of the study. In conclusion, these data suggest that the mechanism by which HGR carbohydrates contributes to obesity is not via prolonged oxidation of the circulating redox state.

## Introduction

Obesity is one of the most pressing contemporary health problems. Despite the wealth of research and the multitude of approaches available to reestablish weight control, the prevalence and severity of overweight and obesity continues to threaten the health of the majority of Americans [1]. Importantly, the cause(s) of obesity are unknown. The diet of the average American has changed significantly in the past several generations and it is hypothesized that it may not be simply the caloric quantity of food consumed, but the composition of the food also that is contributing to the development of obesity and chronic disease [2].

The changes in the structure and composition of Western food are two of the large fundamental changes that have come with the evolution of food technology and consumer demand in the last half a century [3]. This is important because the structure of the food has a direct effect on postprandial hormonal and biochemical responses ([4] and references within). For example, ready-to-eat, processed foods (e.g., frozen pizza, instant mashed potatoes) are increasingly popular and, importantly, commercially processed starches tend to have a higher glycemic response than their conventionally prepared counterparts [5]. Moreover, high blood glucose concentrations cause a vast array of maladaptive physiological and biochemical responses in the body, such as stress to pancreatic islet β-cells [6] and non-enzymatic glycation of proteins [7]. Habitual dietary habits that cause frequent bouts of high blood glucose concentrations can compound these issues, promoting the development of debilitating conditions such as type 2 diabetes [6]. In fact, recent human studies have observed that calorie-restrictive diets that include low glycemic response (LGR) instead of high glycemic response (HGR) carbohydrates, especially in place of saturated fat, is more efficacious for weight loss (reviewed in [8]). Despite the obvious mechanistic linkage between blood sugar fluctuations and metabolic dysfunction, very few controlled research studies have investigated the chronic effects of a HGR diet compared to a LGR diet on the mechanisms of physiological changes in humans or other animals.

There is an established consensus that a HGR diet vs. a LGR diet contributes to increased rate of adiposity, often with insulin resistance, in rodent models [9–22]. There also appears to be a link between a HGR diet and impaired lipid metabolism [13,17,20]. In addition, differences in the glycemic response of the diet results in alteration of gut hormones such as gastric inhibitory polypeptide, glucagon-like peptide-1, GLP-1, and peptide YY [18,21] and leptin, even when correcting for changes in adiposity [19]. Moreover, Belobrajdic *et al*. observed a dramatic, dose-dependent increase in carbohydrate fermentation and production of short chain fatty acids with increasing amounts of a LGR starch in a diet in rodents, suggesting that glycemic response may indeed alter lipid metabolism [18]. HGR and LGR diets can have a plethora of formulations, and the experiments listed above use very different diet formulations and experimental designs to test their hypotheses. Importantly, the presence of dietary fiber in the LGR diet can lead to changes in metabolizable carbohydrate content, which can lead to differential effects on hormones and gut fermentation [23]. However, the fact that thirteen of the fourteen studies concluded that a LGR diet reduces weight gain in the rodents suggests that a broad, all-encompassing signaling mechanism is at play linking the glycemic response to body weight.

We hypothesized that the maladaptive effects of a habitual HGR diet stem from excess reactive oxygen species (ROS) caused by frequent, large fluctuations in blood sugar. To expound, a rapid elevation in blood sugar could lead to a rapid flux of energy through the mitochondrial electron transport chain and produce excess ROS. A frequent pattern of high oscillations in blood sugar and ROS generation could lead to an accumulation of oxidative damage to proteins and lipids. Diet can indeed affect the intra- and extracellular redox state, which has a broad impact on enzymatic and cellular function (reviews: [24–26]). For example, many mechanistic studies have observed that a HGR diet leads to increased insulin resistance, decreased insulin sensitivity, and/or even disruption of islet architecture [11,13,16,17,20,22]. Pancreatic β-cells are particularly susceptible to oxidative stress due to limited antioxidant capabilities [27], and it is possible that excess ROS may explain the consistent link between a HGR diet and dysregulation of insulin signaling. To our knowledge, only two studies have addressed the link between a HGR diet and oxidative stress: Du *et al*. observed a shift of the mouse duodenum towards a more oxidative state after 2 wks on a HGR diet [28], though Coate & Huggins observed no differences in the expression of anti- and pro-oxidant enzymes in adipose tissue between mice fed a HGR or LGR diet [14]. However, with the current data that are available and without an understood underlying mechanism, we decided to further address the link between a HGR diet and ROS.

In order to assure comparability between our study and that of Coate & Huggins [14], we used a similar HGR and LGR diet and experimental design to investigate the effect of a HGR vs. LGR diet on markers of oxidative stress. We assessed longitudinal metabolic measures including energy provision, energy expenditure, and glucose tolerance as well as markers of oxidative stress (e.g., total and oxidized glutathione, lipid peroxidation) after 16 wks on the diets. Additionally, we evaluated the effects of metabolic acclimation to a 4 wk high fat diet while maintaining the starch structure. Based on our hypothesis that long-term consumption of a HGR vs. a LGR diet would lead to oxidation of the circulating redox state, we predicted that a HGR diet would increase fat accumulation, reduce insulin sensitivity, and lead to poorer metabolic acclimation to a high fat diet. Although we observed the mice on the HGR diet gain significantly more adiposity than those on the LGR diet, the HGR diet did not result in greater oxidative stress.

## Materials and Methods

### Materials

Chemicals were purchased from Sigma Aldrich (St. Louis, MO) and general supplies were purchased from Thermo Fisher Scientific (Waltham, MA) unless otherwise noted.

### Experimental design

The experimental design is shown in Table 1. Mice consumed a HGR or LGR diet for 16 wks. Subsequently, half of the cohort consumed a high fat diet (maintaining the HGR or LGR starches) to assess metabolic adaptation. Longitudinal measures included body weight, magnetic resonance imaging (MRI) for body composition, metabolic parameters with metabolic cages, random blood glucose measurements, and an oral glucose tolerance (Table 1). At the end of the study (week 16 or 20), oxidative stress parameters in blood and liver were assessed.

**Table 1:** Experimental design.

Procedure/Week	1	2	3	4	5	6	7	8	9	10	11	12	13	14	15	16	17	18	19	20
Low/high glycemic response diets with 13.4% kcal %	x	x	x	x	x	x	x	x	x	x	x	x	x	x	x	x				
High fat diets (a subset of the mice)																	x	x	x	x
Body weight (3 times/wk)	x	x	x	x	x	x	x	x	x	x	x	x	x	x	x	x	x	x	x	x
MRI^*a*^ (Body composition)				x								x			x					x
Metabolic cages (food and water intake, RER^*b*^, heat production, activity)				x											x					x
Random blood glucose						x							x							
Glucose tolerance test														x						

^*a*^Magnetic resonance imaging

^*b*^Respiratory exchange ratio

### Animal models and experimental diets

This study was carried out in strict compliance with the recommendations in the Guide for the Care and Use of Laboratory Animals. It was approved by the Institutional Animal Care and Use Committee at Boston University School of Medicine (Protocol 2012–15314). Male C57BL/6 mice were purchased at 6–8 weeks old from Charles River Laboratories (Wilmington, MA) and housed in groups of 3 or 4 but were separated if they were fighting. The mice resided in the Laboratory Animal Science Center at Boston University Medical Campus with the lights on between 7:00 am and 7:00 pm daily. The temperature was maintained at approximately 18–24°C and the humidity ranged from approximately 20–80%. Mice were acclimated for 10–14 days before starting on the experimental diet.

Diets were manufactured by Research Diets, Inc. (New Brunswick, NJ) with starches generously provided by Ingredion Incorporated (Bridgewater, NJ). Diet composition was based on the AIN-76A diet (Research Diets, Inc.) and the HGR and LGR diets by Coate & Huggins [14]; they are presented in Table 2. The low glycemic response diet (D12102201) contained Hi-Maize 260 resistance starch which was a corn-derived starch high in amylose (approximately 60%) and approximately 40% amylopectin. The HGR diet (D12102202) contained Amioca waxy maze starch which was 100% amylopectin. For the high fat phase of the experiment, in order to maintain equivalent starch composition as the diets in the first phase, a “very high fat” diet (68.0 kcal %, D13082001) was mixed with a meal form of the HGR and LGR diets at a ratio that yielded 35% kcal from fat. This diet was consumed out of food jars. Mice were fed *ad libitum* for both phases of the experiment.

**Table 2:** Macronutrient composition of the experimental diets.

	Low GR		High GR		High Fat, Low GR		High fat, High GR	
Ingredient	g %	kcal %	g %	kcal%	g %	kcal %	g %	kcal %
Protein	20.3	20.1	20.3	20.1	23.1	20.1	23.1	20.1
Carbohydrate	67.0	66.5	67.0	66.5	51.4	44.8	51.4	44.8
Fat	6.0	13.4	6.0	13.4	17.9	35.1	17.9	35.1
Total	93.3	100.0	93.3	100.0	92.4	100.0	92.4	100.0
kcal/g	4.03		4.03		4.59		4.59	

To confirm that the HGR diet led to a higher glycemic response than the LGR diet, a meal tolerance test was performed with mice who were not part of the main study based on the protocol by Isken *et al*. [12]. Male C57BL/6 mice, about 5–6 months old, were trained for four days by removing food at 9:30 pm, providing 1 g of food at 9:30 am, and then providing excess food from 12:30 pm to 9:30 pm. On day 6, food was removed at 9:30 pm and cages were changed. At 10:30 am on day 7, a baseline blood glucose sample was collected from the tail vein (Bayer Contour blood glucose meter, Whippany, NJ). One hour later, exactly 500 mg of food was provided. Three mice from each group consumed the entire pellet within 5 min. Blood samples were acquired at 15, 30, 60, and 90 min and assessed for blood glucose. The area under the curve (AUC) for change in blood glucose from baseline vs. time was significantly greater for those in the HGR group compared to those in the LGR group (*p* = 0.01, one-tailed *t*-test). Although glycemia had not yet returned to baseline after 90 min, it was decreasing for both diets. Based on these observations and the assumption that our diets lead to similar physiological responses as others [12,14], we concluded that the HGR diet did indeed lead to an overall higher glycemic response than the LGR diet.

In order to quantify the digestibility of the diets, 9 mice, age about 3–4 months old, who were not part of the main study, were used to determine the digestibility of the diets in a randomized, counterbalanced, crossover design. Mice were provided with the LGR or HGR diet for one week; feces were collected every 24 h for the last three days of that week. They were placed on normal chow for one week to washout, and subsequently placed on the other diet for one week, during which feces were collected as in the first phase. Feces were collected from the bedding with tweezers, and dried immediately in a 60°C oven until the weight was stable (at least 24 h). Caloric content was determined using bomb calorimetry at the University of Alabama Birmingham Small Animal Phenotyping Core.

### Longitudinal phenotyping

Fat mass, lean mass, free body water, and total body water were acquired using the 5 G EchoMRI-700 (EchoMRI, LLC, Houston, TX). Mice were assessed between 9:00 am and 12:00 noon in the fed state. The machine was calibrated with a canola oil phantom before every experiment.

Metabolic parameters were determined using indirect calorimetry in metabolic cages (Oxymax Comprehensive Lab Animal Monitoring System, Columbus Instruments, Columbus, OH). Mice were housed individually for 48 h; 24 h for acclimation and then 24 h of data collection. The gas flow rate was 0.5 L/min. The instrument monitored the amount of oxygen inhaled (VO_2_) and the amount of carbon dioxide exhaled (VCO_2_) for about 1 min every 18 min and continuously monitored the amount of activity on x- (long), y- (short), and z- (vertical) axes. Total activity counted the number of times a light beam was broken; ambulatory activity counted the number of times adjacent beams were broken and therefore did not register movement due to breathing, grooming, or scratching. Food and water mass were determined at the beginning and the end of the experiment and intake was determined gravimetrically. Respiratory exchange ratio (RER) was calculated with CLAX software (Columbus Instruments) as VCO_2_/VO_2_. Heat production (or energy expenditure) in cal/min was derived from the Lusk equation: (3.815 + 1.232 × RER) × VO_2_ with VO_2_ in mL/min. The system was calibrated with gas of a known percentage of oxygen and carbon dioxide before every experiment.

Random blood glucose measurements were acquired in the fed state between 3:00 pm – 4:00 pm.

### Glucose tolerance test

An oral glucose tolerance test (OGTT) was performed based on protocols by Andrikopoulos *et al*. and Zhang Lab [29,30]. Briefly, glucose-gelatin tablets were prepared by combining 0.9 g glucose with 1.0 mL water in a glass vial and 0.7 g unflavored gelatin (Knox Original Gelatine, Kraft Foods, Northfield, IL) with 5 mL water in a second glass vial. Solutions were heated on a heating plate at 55°C swirling frequently until all the solid was dissolved (about 10 min). All the glucose was transferred to one well in a 24-well plate. Exactly 650 μL gelatin solution and 150 μL water were placed in the same well and were combined by pipetting up and down. The plate (with lid) was placed at 4°C overnight to allow the gelatin to set. In order to acclimate the mice to the gelatin, approximately 100 mg of gelatin solution was provided to the mice three times the week before the experiment- Monday after an overnight (12 hr) fast, and Wednesday and Friday morning after having food overnight. This training was adequate to train 31 out of the mice for the OGTT. For the OGTT, mice were fasted overnight (10:00 pm–10:00 am). A baseline blood sample was acquired and the mice rested for 30 min. The mice consumed a glucose-gelatin tablet (2 g/kg body weight) within 10 min. Blood samples were acquired at 15, 30, 60, and 90 min after the first bite. For every post-glucose blood glucose measurement, the change in blood glucose was calculated from the baseline measurement and the AUC was calculated using the trapezoid rule [29].

### Tissue harvest

Animal sacrifice was performed with anesthesia methods to minimize pain and suffering of the animals. Mice were fasted overnight (about 12 h). They were anesthetized in an induction chamber with a steady flow of isoflurane (Ohmeda Isotec 3 Vaporizer, Omed of Nevada, LLC, Reno, NV) for 2 min. They were then transferred to the necropsy table while maintaining isoflurane inhalation. Blood was collected via a heart puncture with a 25 G needle; 400 μL of blood was placed in a 1.5 mL centrifuge tube with perchloric acid (PCA) to achieve 20% PCA for metabolite assessment and the rest was placed in a 500 μL EDTA-treated tube (Microtainer, BD, Hunt Valley, MD) for analysis of glutathione, thiobarbituric acid reactive substances (TBARS), and thioredoxin reductase (TrxR) activity. Samples were immediately placed on ice. Plasma was isolated by centrifuging samples at 2000 *g* for 15 min. Liver and adipocyte tissue was placed on pre-weighed pieces of aluminum foil and placed immediately in liquid nitrogen. After weighing the liver samples, they were ground into a fine powder in liquid nitrogen for storage. All tissue samples were stored at -80°C.

### Measures of oxidative stress

Within 48 h of tissue collection, lactate, pyruvate, β-hydroxybutyrate, and acetoacetate were assessed in plasma based on published fluorescent methods [31]. TBARS were assessed in liver and plasma using an assay kit provided by ZeptoMetrix Corporation (Buffalo, NY). Glutathione (total and oxidized) was assessed in liver and plasma using a kit provided by Cayman Chemical Co. (Ann Arbor, MI). TrxR was assessed in liver based on a protocol by Gromer *et al*. [32] that assessed the capacity for TrxR to reduce dithionitrobenzoic acid (DTNB) to thionitrobenzoic acid (TNB). Briefly, liver was homogenized in “T-buffer” (100 mM potassium phosphate and 2 mM EDTA, pH 7.4) in a ratio of 1/5, the sample was centrifuged at 15600 *g* at 4°C, and the pellet was discarded. Samples were diluted as necessary with T-buffer. In a 48-well, clear, flat-bottom plate, the following reagents were combined: 82 μL sample, 3 μL 100 mM DTNB in DMSO, 10 μL T-buffer or 2 mM aurothiomalate inhibitor in T-buffer (to assess reductive capacity of the sample due to agents other than TrxR), and 5 μL 4 mM NADPH in T-buffer. Immediately after adding NADPH, absorbance at 412 nm was read on a microplate reader (Infinite M1000, Tecan Group Ltd., Männedorf, Switzerland) and again after 20 min. A TNB standard curve was created by reducing DTNB to TNB with dithiothreitol (DTT) and plating to yield standards between 0 and 250 μM TNB. One U of enzyme activity is equal to generation of 1 μmol TNB/min.

### Statistics

Data represent the average ± standard error. Statistics were run with JMP (version 10, SAS Institute Inc., Cary, NC). Statistical significance was deemed when *p* < 0.05. The rate of change of body weight was assessed with analysis of covariance with the initial body weight as a covariant. Differences in longitudinal measures (e.g., fat mass, RER) were assessed with an ANOVA assessing the interaction between treatment and time. Differences in end point measures (e.g., TrxR activity) were assessed with a generalized linear model (2 × 2 unbalanced ANOVA) assessing the interaction between fat content and glycemic response of the diet. For heat production data, differences were assessed by normalizing data to both body weight and fat free mass using a random effects ANCOVA [33]. We used a random effects model with a random intercept for the mouse. This accounts for possible correlation between measurements taken on the same mouse. The correlation matrix was produced with R version 3.0.1 (2013-05-16; www.R-project.org). Parameters were included when data were available for at least 14 mice per group (e.g., longitudinal parameters at week 20 were not included because only about half of the mice were subjected to these measurements). In order to determine statistical differences between correlational measures post hoc, the Bonferroni correction was applied conservatively (measures were assumed to be independent, although many were not completely independent). In total, 43 measures were correlated with each other to yield 946 correlations, and a statistically significant *p* value declared at < 0.05/946 = 5.29e-5.

## Results

In order to investigate the effects of a long-term HGR vs. LGR diet on the circulating redox state, metabolic parameters, and metabolic adaptation to a high fat diet in a mouse model, male C57BL/6 mice were fed a diet containing 55% (w/w) waxy maize starch (HGR) or 55% (w/w) Hi-Maize 260 starch (LGR) for 16 wks (Table 2 includes macronutrient breakdown and S1 Table includes a full ingredient list). Then, in order to evaluate metabolic adaptation to a high fat diet, a subset of the cohort consumed a high fat diet with the same starch composition for 4 wks. End point measures included markers of oxidative stress in plasma and liver.

### Mouse health during the experiment

In total, 36 mice were purchased for the study. Several mice presented with genital abscesses during the experiment: *n* = 3 in the LGR group and *n* = 4 in the HGR group. From the LGR diet, 2 were euthanized and 1 had a small abscess at the end of the study; from the HGR group, 1 was euthanized, 2 were treated with lancing and antibiotics and recovered, and 1 had a small abscess at the end of the study. We believe that the abscesses were not related to the treatments.

### Weight gain and longitudinal measures of body composition

**Fig 1:**
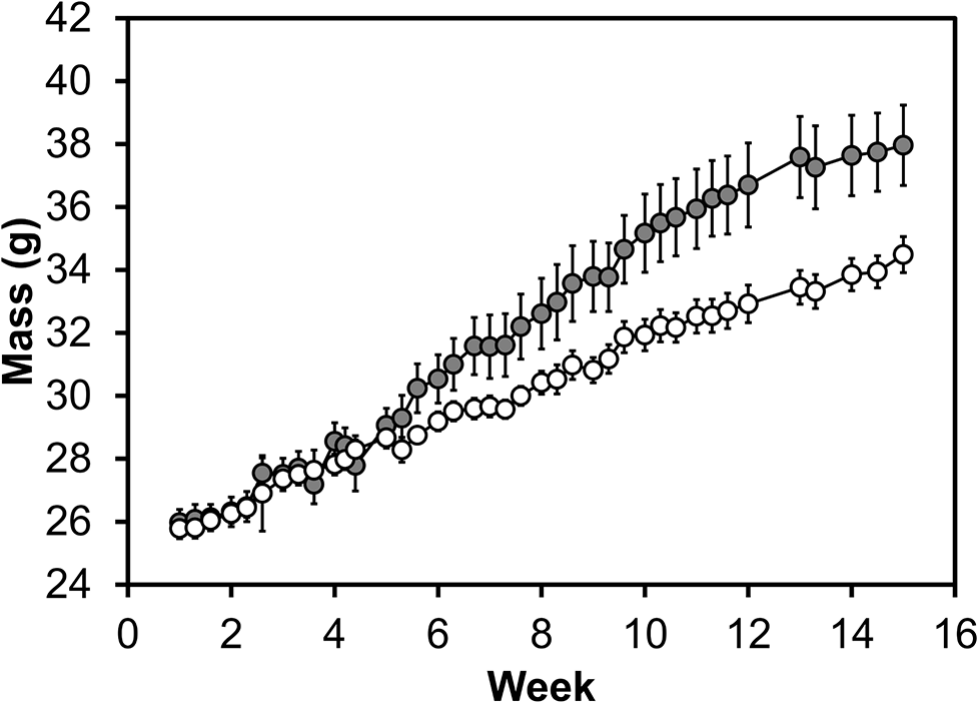
Mouse weight vs. time. Mice on the high glycemic response diet (gray, *n* = 16) gained weight at a faster rate than mice on the low glycemic response diet (white, *n* = 17; *p* < 0.001 for Group, Time, and the interaction Group × Time, ANOVA; data represent avg ± SE).

As expected from previous literature, mice on a HGR diet gained weight at a faster rate than those that were on the LGR diet (Fig 1; *p* < 0.001 for the interaction between Group × Time, ANOVA).

**Fig 2:**
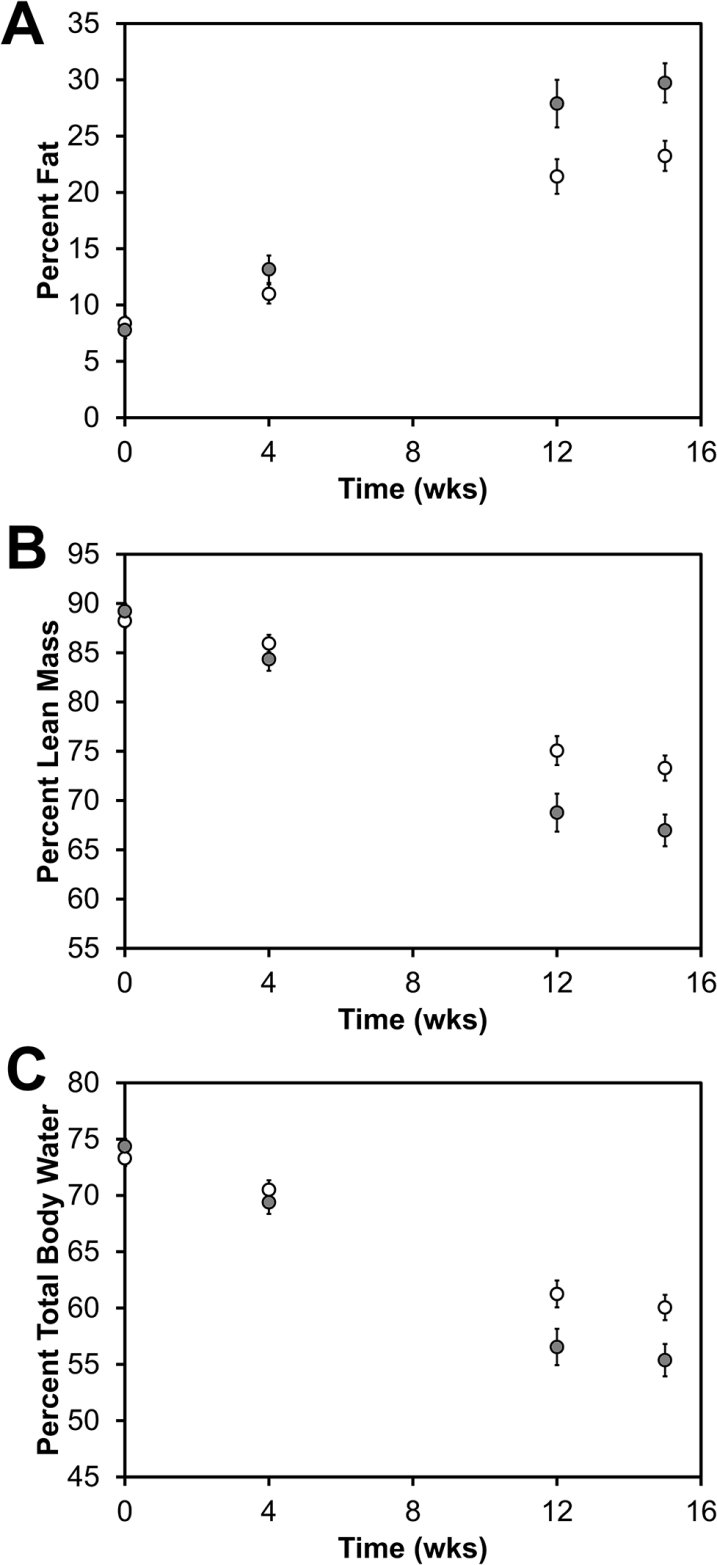
Body composition, as measured by MRI, vs. time. Percent fat mass (A), lean mass (B), and total body water (C) over the first 15 weeks of the experiment. *n* = 17 in the low glycemic response diet group, white, and *n* = 15 in the high glycemic response diet group, gray. There are effects of Group, Time, and Group × Time (*p* < 0.009) for all three measures (ANOVA). Data represent avg ± SE.

Body composition was assessed at weeks 0, 4, 12, and 15 using MRI. Percent fat mass, lean mass, and total body water were similar at baseline (*p* > 0.27; Fig 2). Fat percentage was about 8% at baseline for both groups. Deposition of fat mass occurred at a greater rate in those on the HGR diet compared to those on the LGR diet (*p* = 0.008, ANOVA, Group × Time); after 15 weeks on the diets mice on the LGR diet were 23.2 ± 0.6% fat and those on the HGR diet were 29.7 ± 7.0% fat. Accordingly, percentage of lean mass decreased with time for those on the HGR diet (*p* = 0.004, ANOVA, Group × Time). All mice were approximately 88% lean mass at baseline; 15 wks later those in the HGR group were 67.0 ± 6.4% lean mass and those in the LGR group were 73.3 ± 5.6% lean mass. Percentage of total body water reflected the percentage of lean mass; percentage of total body water in the LGR group decreased at a greater rate than those on the HGR diet (*p* = 0.009, ANOVA, Group × Time).

### Energy provision

**Fig 3:**
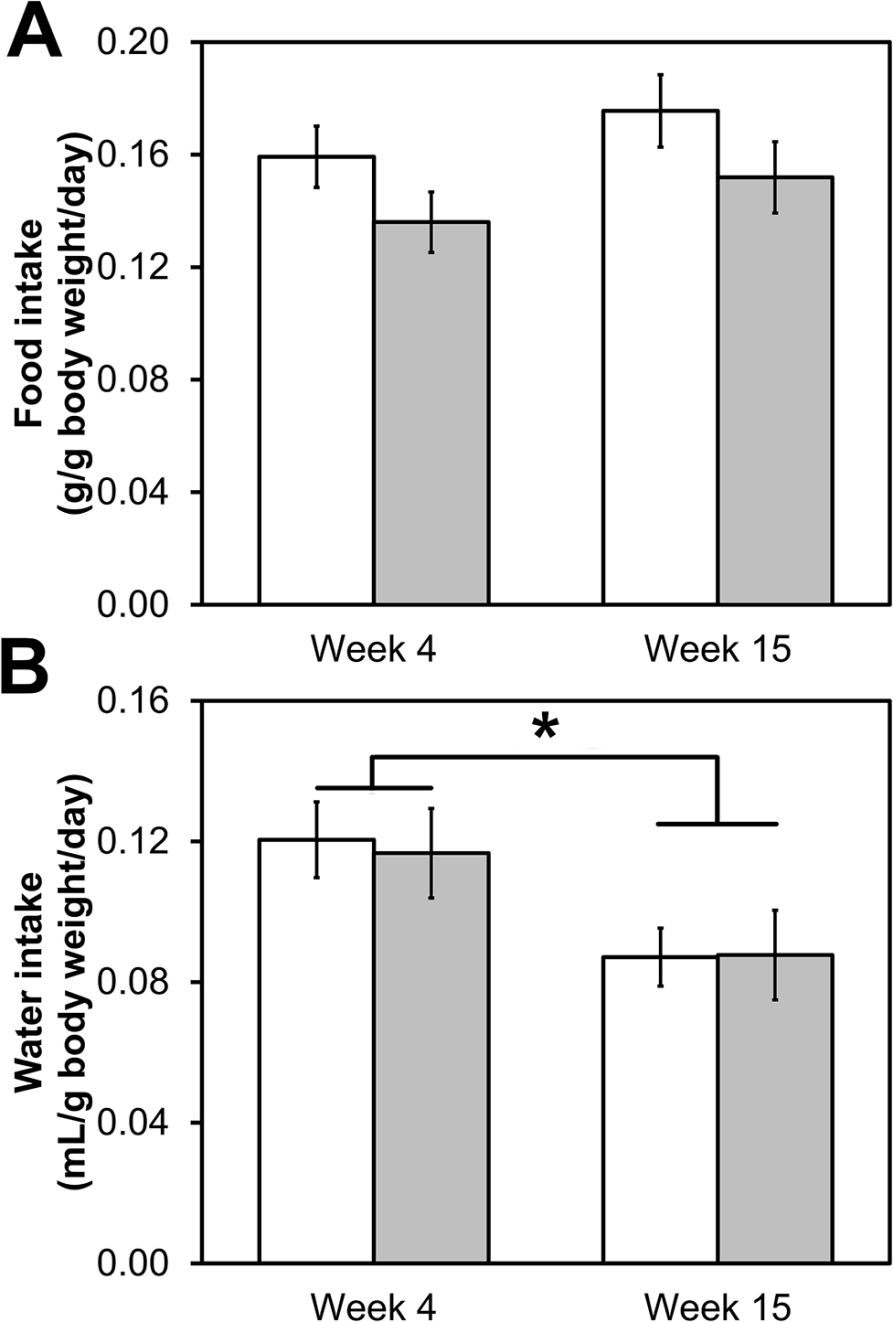
Daily food and water intake at weeks 4 and 15. A) Mice on the low glycemic response diet (white) tended to eat more than those on the high glycemic response diet (gray; *p* = 0.054, repeated measures ANOVA). B) Mice drank less water at week 15 compared to week 4 (*p* = 0.007). *n* = 16 for those on the low glycemic response diet and *n* = 15 for those on the high glycemic response diet; repeated measures ANOVA. Data represent avg ± SE.

In order to determine if the differences in weight gain and body composition result from differences in energy intake and/or energy expenditure, we assessed food intake, water intake, calorie content of excrement, and respiratory parameters. Mice on the LGR diet tended to eat more than those on the HGR diet (*p* = 0.054, repeated measures ANOVA; Fig 3), suggesting that there was lesser digestibility of the LGR diet. Food intake was similar between weeks 4 and week 15 when normalized to total body mass (*p* = 0.18; Fig 3A). Resting energy expenditure [34] and whole body energy expenditure [35] is dependent predominately on fat free mass (FFM) rather than total body mass, and fat mass accumulated at a greater rate than lean body mass. To account for this potential confounder, food intake was also normalized to FFM. Interestingly, food intake was greater at week 15 compared to week 4 (*p* = 0.008; S1 Fig), suggesting that fat mass does indeed contribute to energy demands, even if the contribution is indirect.

In order to assess the energy lost to defecation, mouse feces were assessed for caloric density using bomb calorimetry. The caloric density of feces from mice on the LGR diet was 3.91 ± 0.09 kcal/g whereas the caloric density of the feces from mice on the HGR diet was 3.61 ± 0.05 kcal/g (*p* = 0.007). In addition, mice excreted almost 5 times more feces (dry weight) normalized to body weight on the LGR diet (3.2 ± 1.4 g/kg body weight vs. 0.67 ± 0.30 g/kg body weight, *p* = 0.040). Similarly, Scribner *et al*. observed comparable results; mice on a amylose/amylopectin diet similar to the one used herein (60% amylose) excreted 4.3 times more energy than mice on a pure amylopectin diet [9]. In addition, Isken *et al*. observed similar results with a diet with 70% amylose or pure amylopectin [13]. Therefore, based on energy intake and caloric density of fecal output, the amylose present in the LGR diet was partially resistant to digestion.

Unlike food intake, water intake was similar between groups at weeks 4 and 15 (*p* = 0.89), suggesting that the differences in digestibility of the diet did not affect fluid requirements. When pooling the mice on different diets, the mice drank 0.12 ± 0.05 mL/g body weight/day at week 4 and 0.08 ± 0.04 mL/g body weight/day at week 15 (*p* = 0.007). When water intake was normalized to FFM rather than body weight, however, there were no longer effects of time (*p* = 0.22, S1 Fig). In fact, water intake (mL/g body weight/day) is correlated with FFM percentage (*R* ^*2*^ = 0.2635, *p* < 0.001). From these data it can be concluded that water requirements, but not food intake, may rely more on FFM than total body mass.

### Substrate utilization and energy expenditure

**Fig 4:**
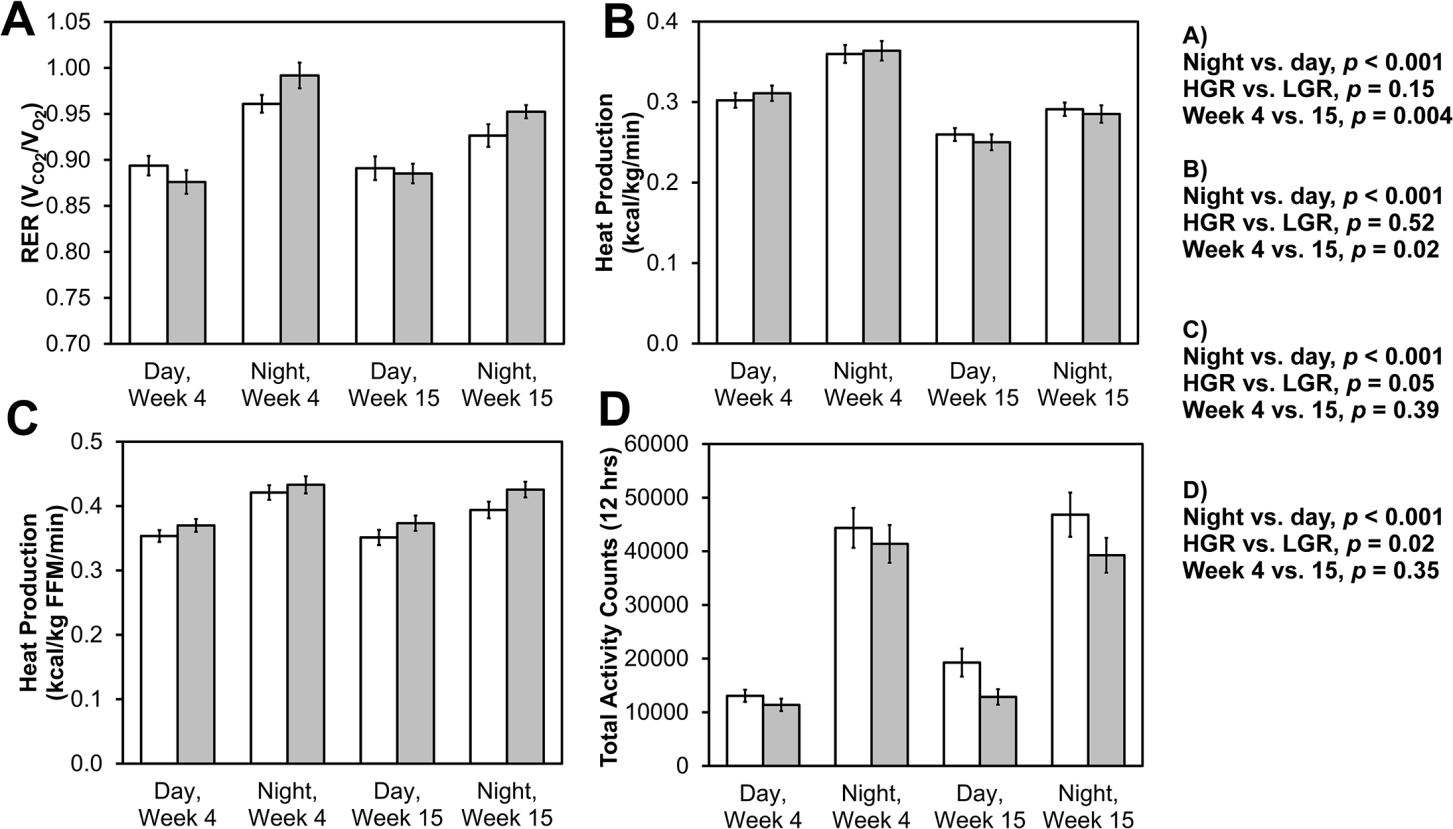
Metabolic parameters during the light and dark cycles at weeks 4 and 15. A) The respiratory exchange ratio (RER), B) Heat production (or total energy expenditure) normalized to body mass, C) Heat production normalized to fat free mass (FFM), and D) The total activity counts for day and night periods for mice on the low (white) and high (gray) glycemic response diets at night (dark cycle) and during the day (light cycle) for weeks 4 and 15 of the diet. *n* = 16 on the high glycemic index diet and *n* = 16 on the low glycemic index diet. Data represent avg ± SE.

The RER is used to determine the provision of energy from carbohydrates or fat, where an RER of 1.0 suggests that energy is being derived from carbohydrates (6 carbon dioxide molecules/6 oxygen molecules) and an RER of 0.7 indicates that energy is being provided by fat (16 carbon dioxide molecules/23 oxygen molecules for palmitic acid). Mice had a greater RER at night vs. during the day (Fig 4A, *p* < 0.001), as expected from their nocturnal eating behaviors and subsequent carbohydrate utilization. In addition, RER was slightly less at week 15 vs. week 4 (*p* = 0.004), suggesting greater turnover of lipid stores in the fatter mice at week 15. Mice on the HGR diet tended to have a greater RER (*p* = 0.15), consistent with lower carbohydrate availability in the LGR diet, but perhaps also due to impaired fat oxidation. By comparison, Isken *et al*. observed a significant delay in the shift in macronutrient provision in mice routinely consuming a HGR diet when mice were fasted and then re-fed [13]. They attributed these observed changes in glucose metabolism to changes in body composition but impairment in fat oxidation to physiological changes brought on by the HGR diet [13].

Heat production (or energy expenditure) by the mice in the HGR and LGR diets at weeks 4 and 15 during the day and night cycles is shown in Fig 4B and 4C. When normalized to total body mass, the glycemic response of the diet did not affect heat production (*p* = 0.52), though heat production was greater during the night (*p* < 0.001) and greater at week 4 vs. week 15 (*p* = 0.02; Fig 4B). Because fat mass contributes only trivially to energy expenditure, it is sometimes prudent to normalize to FFM instead of total body mass [35]. Indeed, the effect of time disappeared when normalizing heat production to FFM (*p* = 0.39; Fig 4C). Interestingly, when normalized to FFM instead of total body mass, mice on the HGR diet tended to exhibit greater heat production than those on the LGR diet (*p* = 0.052). It is not expected that heat production would be different if differences resulted solely from the changes in body composition, so this result ought to be validated before it is extended upon.

### Activity

Activity was measured for 24 h after an acclimation period in metabolic cages at weeks 4 and 15. Total activity counts, which take into account ambulatory activity as well as scratching, grooming, feeding, and fidgeting, are displayed to 1 min resolution in Panel A in S2 Fig (week 4) and Panel B in S2 Fig (week 15). Total activity counts were pooled for the 12 h diurnal cycles and presented as the mean ± SD in Fig 4D. Mice on the LGR diet partook in slightly but statistically significantly more activity than those on the HGR diet (*p* = 0.02, generalized linear model). As expected, nocturnal activity was significantly greater than diurnal activity (*p* < 0.001). There were no differences in activity between weeks 4 and 15 (*p* = 0.35).

Ultradian rhythms, or behavioral rhythms that are less than a 24 h period, were empirically suggested by the raw activity curves with a period circa 1–2 h (Panels A and B in S2 Fig). If the HGR diet required more frequent feedings, we may expect ultradian rhythms to have a shorter period for mice on the HGR diet. In an exploratory analysis, we filtered out rhythms from the day/night cycle using a high pass filter (removed cycles with a period >4 h) and then used maximal entropy spectral analysis (MESA) to mathematically assess the presence of ultradian rhythms with a period of about 1–2 h [36] (see S3 Fig for more information). Mice on the LGR diet exhibited the strongest locomotive oscillations at 1.73 h at week 4 and 1.46 h at week 15; mice on the HGR diet showed the strongest oscillations in activity at 1.18 h at week 4 and then 1.46 h at week 15 (S3 Fig). Therefore, oscillations in activity occurred with higher frequency in mice on the HGR diet at week 4 but not week 15. More research is necessary to determine if oscillations in activity are physiologically and/or behaviorally relevant, and if the structure of the diet can affect *ad libitum* feeding frequencies.

### Blood glucose

Random blood glucose measurements are useful for obtaining a “snapshot” of circulating blood glucose levels in an unfasted human or animal. A random blood glucose concentration of <150 mg/dl is typically considered healthy, while a random blood glucose concentration of >150 mg/dl indicates potential diabetes in humans [37], and these thresholds are often used in rodents as well [38]. Random blood glucose measurements were obtained at weeks 6 and 13 between 3:00pm and 4:00 pm. At week 6, mice on the LGR diet exhibited a random blood glucose measurement of 118 ± 17 mg/dl (mean ± SD) and mice on the HGR diet exhibited a random blood glucose measurement of 115 ± 32 mg/dl (*p* = 0.70, *t*-test). At week 13, mice in the LGR group had a random blood glucose measurement of 112 ± 18 mg/dl and those on the HGR diet had a random blood glucose measurement of 117 ± 18 mg/dl (*p* = 0.50). These data suggest that mice in both groups were normoglycemic and that the HGR food did not lead to prolonged elevations in blood glucose.

In order to more fully evaluate glucose handling, an OGTT was performed at week 14. In order to simulate postprandial physiology, mice were trained to voluntarily consume 2 g glucose/kg body weight in the form of a gelatin tablet [30]. Fasting blood glucose did not differ between diet groups (94 ± 14 mg/dl in the LGR diet group and 92 ± 20 mg/dl in the HGR diet group; *p* = 0.75, *t*-test). In response to the OGTT, neither maximum blood glucose (214 ± 29 mg/dl for the LGR group and 209 ± 24 for the HGR group, *p* = 0.55) nor area under the curve for the 90 min experiment (6192 ± 2050 min·mg/dl for the LGR group and 6850 ± 1315 min·mg/dl for the HGR group, *p* = 0.30) differed between groups. These data indicate that 14 weeks on the HGR vs. LGR diet did not lead to differences in glucose tolerance or apparent insulin sensitivity.

### Metabolic acclimation to a high fat diet

It has been well established that individuals vary greatly in their accumulation of adipose tissue in response to changes in the macronutrient content of the diet [39]. Even *et al*. coined the term “adiposity sensitivity” to describe the inclination to gain fat mass in response to a dietary perturbation [39]. In this experiment, after 16 weeks on the LGR or HGR diets, the fat was increased in the diet from 13.4% to 42.7% of kcal (Table 1) with Primex shortening for a subset of the cohort while maintaining the starch composition. They consumed the diets for 4 weeks. During the last week, the mice were assessed for body composition using MRI and respiratory parameters using metabolic cages. They were then sacrificed in an equivalent manner as the mice that were not subjected to the high fat diet.

During the 4 weeks on the high fat diet, the mice consuming LGR or HGR starch gained weight at an equal rate (*p* = 0.58 for the effect of diet, ANOVA; Panel A of S4 Fig) and did not differ in body mass at the end of the experiment (*p* = 0.65). Between weeks 16 and 20, the mice on the LGR diet increased from 23.6 ± 6.9% fat to 34.9 ± 4.3% fat and the mice on the HGR diet increased from 26.2 ± 6.5% fat to 32.9% fat with no difference between groups at week 20 (*p* = 0.40). Percent FFM decreased accordingly during this leg of the experiment with no difference between groups (64.0 ± 3.6% FFM for mice on the HGR diet and 63.1 ± 3.9% FFM for mice on the LGR diet at week 20, *p* = 0.61). Similarly, percent total body water was similar between groups at week 20 (53.3 ± 3.6% for those on the HGR diet and 52.4 ± 3.6% for those on the LGR diet, *p* = 0.58).

There were no differences in water consumption between diet groups when normalizing to total body mass (0.060 ± 0.016 mL/g/day for LGR and 0.067 ± 0.025 mL/g/day for HGR, *p* = 0.44) or lean body mass (0.095 ± 0.024 mL/g FFM/day for LGR or 0.104 ± 0.033 mL/g FFM/day for HGR, *p* = 0.51). However, the mice consumed less water at week 20 compared to week 15 when normalizing to total body mass (*p* < 0.001) or lean body mass (*p* = 0.004).

The RER of the mice at week 20 is shown in Panel B in S4 Fig. The RER was much lower for the mice on the higher fat diet compared to on the lower fat diet due to the greater provision of energy from fat (RER about 0.80 compared to 0.85–1.0, Fig 4). Interestingly, the RER was not greater during the night as it was in the first leg of the study (*p* = 0.40). However, the greater RER for the mice consuming the HGR starch was retained (*p* = 0.035), likely due to the greater digestibility of the starch. The RER and the food quotient are similar after adaptation to a diet, though it was not possible to calculate the food quotient in this study because the indigestibility of the starch could only be roughly estimated. In future studies, it would be fruitful to assess the food quotient and its relation to the RER in order to assess whether the mice have adequately adapted to the new diet.

Heat production (energy expenditure) during week 20 is shown in Panel C in S4 Fig. Heat production, when normalized to total body mass was significantly greater at night vs. during the day (*p* = 0.002), yet there were no differences between diet groups (*p* = 0.61). Similarly, when normalized to FFM, heat production was greater during the night (0.39 ± 0.06 kcal/kg FFM/min) vs. day (0.33 ± 0.04 kcal/kg FFM/min, *p* = 0.001) yet there were no differences between diet groups (*p* = 0.94).

For metabolic cage experiments with the high fat diets, food was provided during the metabolic cage experiments in food jars. The food jar occupied a large proportion of the floor area and the mice were often found burrowing and pushing the jar around; hence, it was not possible to obtain reliable measurements for food intake, total activity, or ambulatory activity during this experiment.

### Post-mortem redox measures

**Fig 5:**
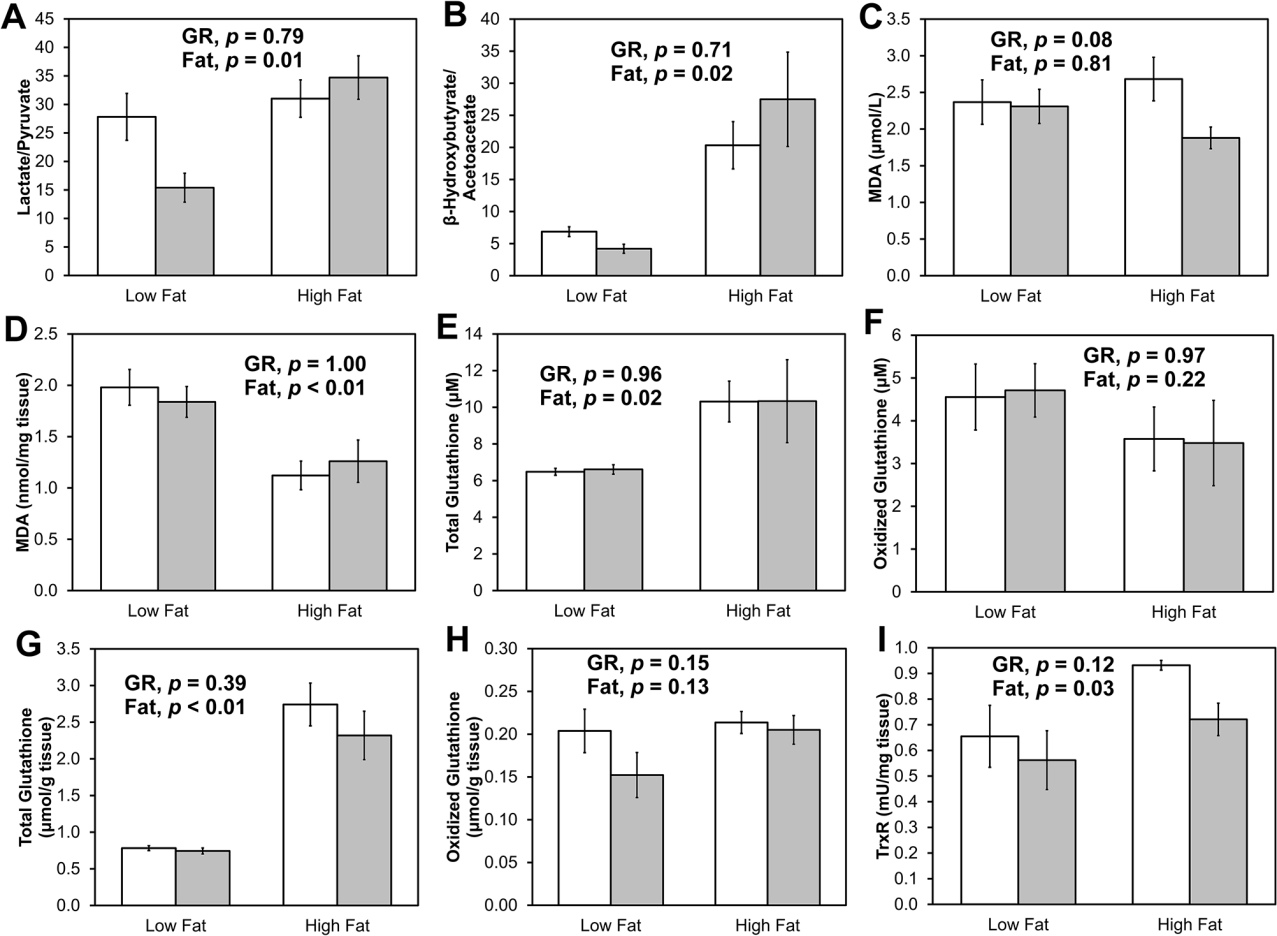
*Ex vivo* redox measures. At the termination of the study, blood and liver were harvested for redox analysis. Mice that were on the low glycemic response diet are in white and those on the high glycemic response diet are in gray. Mice that were terminated during the 16^th^ week are labeled “Low Fat” while those subjected to the high fat diet for 4 weeks succeeding the low fat leg are labeled “High Fat”. Data represent avg ± SE. A) Lactate/Pyruvate ratio; B) βOHB/Acoc ratio; C) TBARS- blood; D) TBARS- liver; E) Total glutathione- blood; F) Oxidized glutathione- blood; G) Total glutathione- liver; H) Oxidized glutathione- liver; I) Thioredoxin reductase activity in liver.

In order to determine if the LGR vs. HGR diets affected markers of oxidative stress with or without subjection to a high fat diet for 4 wks, a battery of assays was performed at the end of the study (Fig 5). After an overnight fast, mice were anesthetized under inhaled isoflurane and tissues were extracted.

The ratio of lactate/pyruvate (L/P) in serum reports the cytosolic NADH/NAD^+^ ratio, while the serum ratio of β-hydroxybutyrate/acetoacetate (B/A) reports the mitochondrial NADH/NAD^+^ ratio [25]. The glycemic response of the diet did not affect serum L/P (Fig 5A; *p* = 0.79) or B/A (Fig 5B; *p* = 0.71). However, those subjected to the high fat diet had a 1.5-fold greater L/P (*p* = 0.01) and a 4-fold greater B/A (*p* = 0.02). This suggests either more reduced cytosolic and mitochondrial redox states or greater availability of NAD(P)H.

TBARS is a measure of lipid peroxidation. TBARS was not different between HGR and LGR groups in plasma (Fig 5C; *p* = 0.08) or liver (Fig 5D; *p* = 1.00). Interestingly, those subjected to the high fat diet had less peroxided lipid in liver than those who did not consume the high fat diet (high fat: 1.2 ± 0.5 nmol/mg tissue, low fat: 1.9 ± 0.4 nmol tissue, *p* < 0.01), though differences were not seen in blood (*p* = 0.81). There was no interaction between fat and glycemic response in the blood (*p* = 0.13) or the liver (*p* = 0.41).

Neither total nor oxidized glutathione concentrations in plasma or liver were affected by the glycemic response of the diet (Fig 5E–5H; *p* > 0.23). Interestingly, total levels of glutathione were elevated in the mice who consumed a high fat vs. normal fat diet (*p* ≤ 0.01 in plasma and liver), though the amount of oxidized glutathione was not different between high and low fat conditions (*p* > 0.13 in blood and liver).

TrxR reduces thioredoxin, which regulates many redox-sensitive enzymes via reducing cystine bonds. There was no difference between the HGR and LGR diets (Fig 5I; *p* = 0.12), though those who consumed the high fat diet exhibited slightly elevated TrxR activity (*p* = 0.03).

Collectively, these data show that the HGR vs. low LGR diets did not affect circulating redox state or plasma or hepatic markers of oxidative stress. Similarly, Coate & Huggins [14] observed no differences in mRNA expression of catalase, glutathione peroxidase, or superoxide dismutase in the epididymal adipose tissue of mice at the end of a 16-week HGR vs. LGR diet study. However, 4 weeks on the high fat diet led to increased total glutathione in plasma and liver, increased hepatic TrxR activity, increased L/P and B/A ratios, and reduced lipid peroxidation, irrespective of the type of starch in the diet. These data suggest that the increased fat availability via the diet could increase reducing equivalents, which, together with increased scavenging, could help control ROS.

### Correlational measures

**Fig 6:**
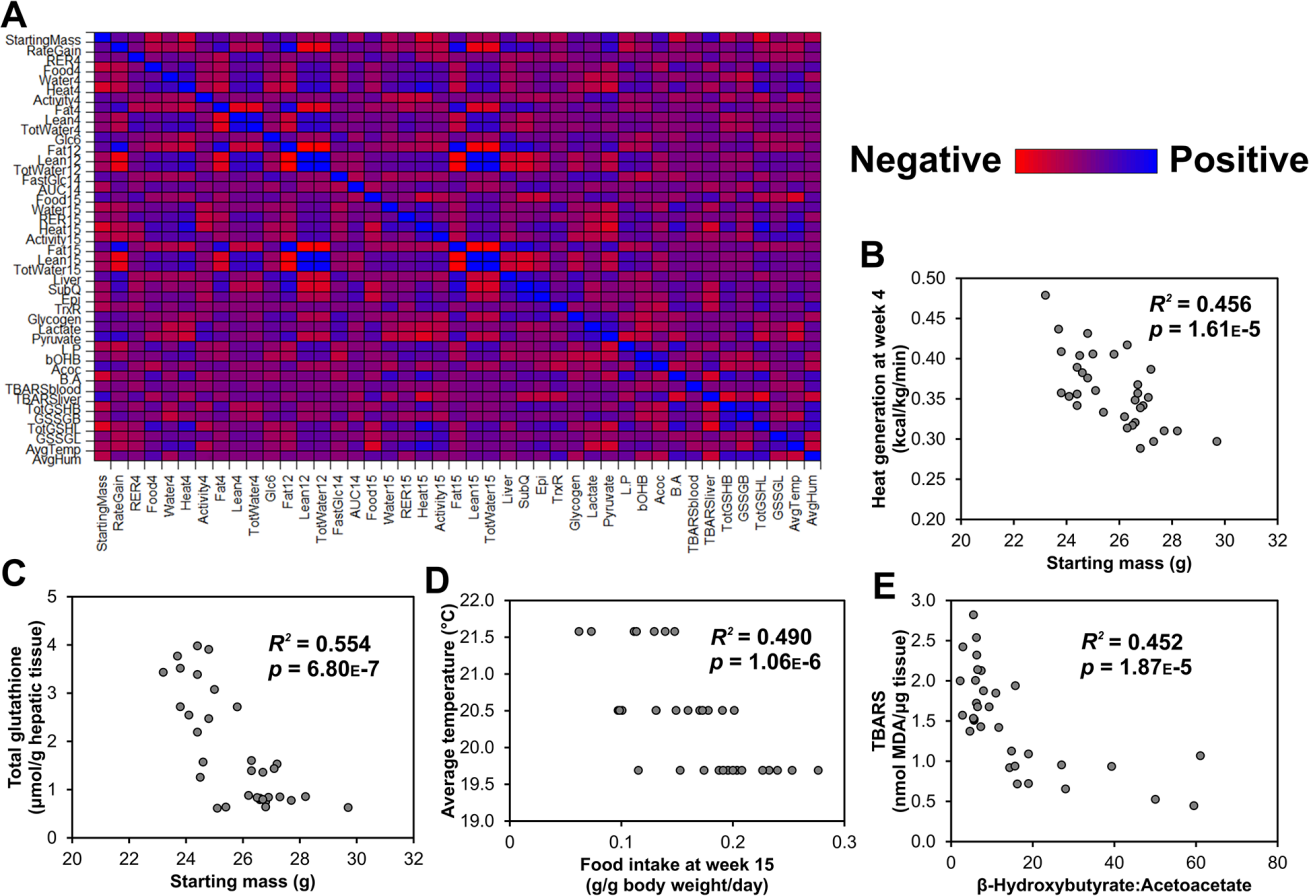
Correlations between baseline, longitudinal, and end point measures. A) Correlation matrix chromatically depicting correlations between all parameters. Blue indicates a positive correlation (*r* = 1) and red indicates a negative correlation (*r* = -1). B) Correlation between heat generation (energy expenditure) during the dark cycle at week 4 and the mouse starting mass, C) Correlation between total glutathione in the liver at the termination of the study and the starting mouse, D) Correlation between the average temperature in the housing facility and food intake at week 15, E) Correlation between TBARS (lipid peroxidation) in the liver and the β-hydroxybutyrate:acetoacetate (B/A) ratio in the serum, both at the end of the study. Starting mass = Mass at the start of the study; RateGain = the rate at which weight was gained during the study; RER4 = average resting energy expenditure during the metabolic cage experiment during 24 hrs during week 4 (kcal/kg/min); Food4 = Food consumed during the fourth week of the experiment (g/g body weight/day); Water4 = Water consumed during the fourth week of the experiment (mL/g body weight/day); Heat4 = average heat generated during the metabolic cage experiment during the dark cycle during week 4 (kcal/kg/min); Activity4 = Total x, y, and z beam breaks during 24 hrs in the metabolic cages; Fat4 = percent fat, as measured by MRI, at week 4; Lean4 = percent fat, as measured by MRI, at week 4; TotWater4 = percent total water, as measured by MRI, at week 4; Glc6 = random blood glucose measurement at week 6; Fat12, Lean12, and TotWater12 = body composition as measured by MRI at week 12; FastGlc14 = fasting blood glucose concentration at week 14; AUC14 = area under the curve of a blood glucose vs. time plot during a glucose tolerance test at week 14; Food15, Water15, RER15, Heat15, Activity15 = metabolic measures during the metabolic cage experiment during week 15 as in week 4; Fat15, Lean15, TotWater15 = body composition as measured by MRI at week 15; the following measures are post-mortem: Liver = liver mass at the end of the study; SubQ = mass of subcutaneous fat mass at the end of the study; Epi = mass of epididymal fat mass at the end of the study; TrxR = thioredoxin reductase activity in the liver; Glycogen = glycogen content of the liver; Lactate = serum lactate concentration; Pyruvate = serum pyruvate concentration; L:P = serum lactate:pyruvate ratio; bOHB = serum β-hydroxybutyrate ratio; Acoc = serum acetoacetate ratio; B:A = serum β-hydroxybutyrate:acetoacetate ratio; TBARSblood = TBARS (lipid peroxidation) in the blood; TBARSliver = TBARS (lipid peroxidation) in the liver; TotGSHB = total glutathione concentration in the blood; GSSGB = oxidized glutathione concentration in the blood; TotGSHL = total glutathione concentration in the liver; GSSGL = total oxidized glutathione concentration in the liver; AvgTemp = average temperature in the facility during the entire experiment; AvgHum = average humidity in the facility.

There were large individual differences between mice in regard to weight gain, metabolic parameters, and ROS scavenging abilities. Therefore, to assess further insight into how some of these parameters may be related, correlations were assessed between baseline, longitudinal, and end-study measures that included the majority of the mice (Fig 6). In total, 946 correlation coefficients were calculated and, using the Bonferroni correction, a statistically significant *p* value declared at < 0.05/946 = 5.29e-5; 46 correlations were statistically significant (S2 Table). Many of the correlations were expected; for example, though measured independently with MRI, fat and lean mass percentage show a strong negative correlation at weeks 4, 12, and 15 (*p* < 1.00e-5, uncorrected). Similarly, subcutaneous fat mass was positively correlated with epididymal fat mass at the end of the study (*p* = 1.42e-7, uncorrected). Additionally, there were several unexpected correlations that lend insight into how parameters may be related including innate metabolic parameters, environmental conditions, redox scavenging ability, and energy balance. First, starting mass was negatively correlated with heat generation (i.e., energy expenditure) during the dark cycle 4 weeks into the study (Fig 6B; *R* ^*2*^ = 0.456, *df* = 31, *p* = 1.61e-5) implying that innately larger mice exhibit less energy expenditure per gram body weight, and that their energy production may be more efficient. (When energy expenditure is normalized to FFM instead of body weight the relationship is maintained; *R* ^*2*^ = 0.465, *p* = 1.25e-5). However, because body weight at 4 wks is correlated with body weight at baseline, these results may stem from differences not in metabolism but in body composition. Total glutathione in hepatic tissue at the termination of the study was also negatively correlated with starting mass of the mice (Fig 6C; *R* ^*2*^ = 0.554, *p* = 6.80e-7). This implies that innately smaller mice may generate less ROS or may be more efficient at scavenging ROS via the glutathione system. Fig 6D shows a correlation between the food intake at week 15 and average room temperature of the animal facility during the experiment (mice were recruited in three cohorts to simplify data collection). An elevated average temperature in the facility was correlated with less food intake (*R* ^*2*^ = 0.490, *p* = 1.06e-6), implying that a colder environment, *on average*, encourages greater food consumption. Interestingly, lipid peroxidation in the liver was negatively correlated with the B/A ratio in the serum at the termination of the study (Fig 5E; *R* ^*2*^ = 0.452, *p* = 1.87e-5). As B/A is typically a reporter of the mitochondrial redox state [25], it may be expected that a greater B/A would associate with greater fat oxidation and ROS scavenging ability due to a greater amount of β-hydroxybutyrate that infers a greater availability of NAD(P)H available to scavenge ROS.

## Discussion

### Mice on the HGR diet gained fat mass at a faster rate, though did not appear to have greater signs of oxidative stress

An increase in adiposity with or without an increase in body weight is often observed in long-term glycemic response experiments in rodents (Fig 1, [9–21]), though not in all studies [22]. The most widely cited hypotheses are hormone/behavior based, noting that 1) a slow yet sustained introduction of glucose to the blood regulates gastrointestinal hormones, such as incretins, that help regulate appetite and peripheral glucose metabolism, and 2) fermentable fiber, which is typically associated with a reduced and sustained glycemic response, can increase short chain fatty acid formation in the colon and stimulate appetite-suppression hormone secretion (reviews: [23,40]). Molecular observations afford greater understanding of biochemical mechanisms: 1) a HGR diet impaired insulin-stimulated glucose oxidation and increased glucose incorporation into lipid in adipocytes [20]; 2) a HGR diet stimulated fat storage and fatty acid synthesis via upregulation of genes related to fatty acid metabolism in the liver [13]; 3) viscous dietary fiber modulated PGC-1α activity and activated AMPK, thereby increasing fatty acid oxidation and mitochondrial biogenesis in muscle [41]; 4) short chain fatty acids from microbial starch fermentation increased fat oxidation [42] and improved glucose uptake into skeletal muscle [43]. However, a complete understanding of these complex, multifaceted interactions remains distant.

Though it has not been shown explicitly in rodents, HGR diets are generally believed to induce post-prandial hyperglycemia after most meals. Glucose fluctuations appear to trigger oxidative stress to a greater magnitude than constantly elevated blood glucose in humans [44]. Both in this study (Fig 5) and in the study by Coate & Huggins [14], markers of oxidative stress were not exacerbated in mice fed the HGR diet compared to the LGR diet. A major limitation in the design of these studies is that food was provided *ad libitum*, yet mice eat frequently throughout the light and dark cycles [45]. In future long-term glycemic response studies with rodents, continuous glucose monitoring [46] is warranted to assess whether the HGR of the feed does indeed result in greater blood glucose fluctuations than the LGR feed. Alternatively or additionally, meal times could be regimented to mimic humans’ eating behaviors.

Mice on the HGR diet likely experienced changes in lipid metabolism enzymes over the course of the study. Indeed, Isken *et al*. [13] observed increased expression of genes related to lipogenesis in the liver after exposure to a HGR diet. In addition, van Schothorst *et al*. [11] observed elevated expression of leptin and lower expression of resistin and adiponectin in mice fed a HGR diet. Future studies assessing gene expression of lipid metabolism genes as well as the localization of fat deposition (i.e., in the muscle) may be important in elucidating the link between fat accumulation and insulin resistance.

### Metabolic adaptation to a high fat diet led to greater efficiency at dealing with oxidative stress

Metabolic flexibility is a term that is used to describe the agility of a metabolic system to switch between carbohydrate and fat oxidation, depending on the substrate availability. Inflexibility in the switch between macronutrient oxidation on the time scale of hours, for example between pre- and postprandial states, has been linked with metabolic syndrome [47]. Isken *et al*. [13] observed that mice on a HGR diet had decreased metabolic flexibility in a 5 h window after a meal, meaning that they switched from carbohydrate to fat oxidation slower than mice that were on a LGR diet. However, it was unknown whether or not this immediate, post-prandial metabolic inflexibility imposed by the HGR diet would lead to greater sensitivity to the high fat diet in regard to increased adiposity. Despite sensitivity to the carbohydrate type, there was no difference in weight gain or noticeable deterioration in metabolic health between the mice that were fed the LGR vs. HGR when subjected to a high fat diet for 4 wks (S4 Fig). Moving forward, it will be important to evaluate the potential for a 5 h metabolic flexibility experiment to serve as a diagnostic test for adiposity sensitivity.

High-fat-induced oxidative stress is a well-established phenomenon [48] and is integral to a model on which antioxidants are tested for efficacy. Herein, mice that were subjected to the high fat diet for 4 wks after the 16 wk HGR/LGR leg of the study exhibited fewer markers of oxidative stress (i.e., less lipid peroxidation in the liver, more total glutathione in the blood and liver, greater hepatic TrxR activity) and the suggestion of greater availability of reducing equivalents (i.e., greater availability of NADH will push the equilibrium towards lactate and βOHB about lactate dehydrogenase and βOHB dehydrogenase, respectively; Fig 5). It is unknown why the mice were not afflicted with increased oxidative stress upon exposure to the high fat diet. One potential explanation for the discrepancy is that diet-induced obesity and associated oxidative stress is often achieved with a diet that delivers 60% of calories from fat [48]. This high amount of fat is not physiological, and perhaps results in much more metabolic stress than the 35.1% kcal from fat diet used herein. Moreover, it is unknown whether extending the high fat feeding period beyond 4 weeks would result in differences between the groups. Matsuzawa-Nagata et al. [49] observed drastic changes in oxidative stress after mice consumed a high fat diet for 6 weeks. The fat source is also an important consideration. The fat source used herein was shortening (mostly oleic acid (37.2%), elaidic acid (22.9%), and palmitic acid (16.4%)) and did not have added preservatives or antioxidants. Also, a potential confounding factor of this diet is that it was delivered in meal form rather than pellet form in a food jar. For this reason, unfortunately, activity levels were not able to be quantified in the metabolic cages due to the presence of the food jar, but it is thought that the mice performed much more activity while digging in the food and grooming, because they got food all over their bodies while digging in the jars. An increase in activity could have led to an increase in glucose utilization in the skeletal muscle, thereby counteracting deleterious effects of the high fat diet on oxidative stress parameters.

We suspect that the mice in this study were particularly apt to controlled nutrient partitioning. Mice often [9,11,13,16,17,22], but not always [18], develop insulin resistance with HGR diet-induced obesity, though these mice remained insulin sensitive after 14 wks of the HGR diet. Subsequently, upon exposure to the high fat diet, the mice burned the fat for energy, as evidenced by the lower RER (Fig 4A vs. Panel B of S4 Fig) and less total lactate + pyruvate at the end of the study. A habitual high fat diet leads to an upregulation of pyruvate dehydrogenase kinase (PDK) in skeletal muscle, a major regulator of fuel partitioning [50]. Pyruvate dehydrogenase (PDH) converts pyruvate into acetyl-CoA and carbon dioxide with NAD^+^ as an electron acceptor. The acetyl-CoA is then shuttled into the TCA cycle to generate more reducing equivalents or used for fatty acid or cholesterol synthesis. When PDH is phosphorylated by PDK, PDH becomes inactive, thereby preventing pyruvate from entering the mitochondria and preventing glucose oxidation. The excess reducing equivalents perhaps increased the mitochondrial leak at first, and compensatory mechanisms were engaged to fight the additional oxidative stress from the diet. In addition, high NADH can be converted to NADPH via the transhydrogenase (NNT) using the proton gradient, which generates a leak. It is unknown whether the high fat diet invoked inflammation or insulin resistance in these 4 weeks; in depth analysis of skeletal muscle lipid content, activity of metabolic enzymes, and redox markers will be important in resolving this discrepancy.

### Correlation between various parameters lends insight into the mechanisms of weight regulation

Some humans respond more readily to dieting and/or exercise than others, and there is a strong initiative to identify predictors of success early in the intervention in order to prevent discouragement, wasted time, and wasted money [51]. Genetic, behavioral, and psychological factors have been the focus of many studies, yet these data suggest that there is great potential for biochemical biomarkers as either predictors of adiposity sensitivity or diagnostic tools. The fact that the mice in this study were genetically similar and reared in an environment as similar as possible in combination with the large inter-individual variability in measures calls for more thorough analysis of metabolic and oxidative stress parameters *before* the initiation of a dietary intervention. Herein, baseline data regarding metabolic parameters and innate antioxidant defenses could have proved valuable. Indeed, simply the starting mass of the mice was correlated with heat production/energy expenditure at week 4 and total glutathione levels at the end of the study, which suggests that the mice have innate propensities to metabolic regulation that 1) may be difficult to perturb with environmental interventions, though 2) once understood could be harnessed to efficiently and effectively improve metabolic health.

### Final remarks

A potential confounding factor in the macronutrient design of this study is that the amylose used in the LGR diet was not completely digestible. The resistance to gastrointestinal murine enzymes led to increased food consumption (g/g, Fig 3) and therefore an increase in the other components of the food (e.g., protein, vitamins) in order to garner adequate energy as well as a drastic change in the gut microflora. We are not certain if the differences in nutrient intake led to differences in metabolism, and future studies ought to strive for properly matched digestible macronutrients rather than total macronutrients. Indeed, the observation that heat production/energy expenditure was lower in the mice on the LGR diet when normalized to FFM (Fig 4C) could be a result of anaerobic energy gleaned from gut microbes that was overlooked by the indirect calorimetry methods [35]. Many other studies have used a partially resistant starch in their LGR diet (e.g., [9]), and the presence of the resistant starch may be physiologically relevant in a human’s LGR diet.

The mechanisms by which a HGR diet leads to metabolic dysfunction are important so that nutritionists, food companies, policy makers, and pharmaceutical companies can collaborate to minimize the effects of HGR food on weight gain. Herein, we observed that a HGR vs. LGR diet did not affect the circulating redox state, as measured by ratios of redox pairs (L/P and B/A), hepatic TrxR activity, total and oxidized glutathione in blood and liver, and lipid peroxidation in blood and liver. Future research is required to glean insight into how a HGR diet, among other aspects of the modern diet, results in increased adiposity and reduced insulin sensitivity. Also, people tend to consume varied diets on a day-to-day basis; it would be interesting to explore whether acclimation to a high fat or HGR diet would attenuate the metabolic stress of a single high fat or HGR meal. The etiology of obesity and obesity-related co-morbidities are unknown, and elucidation of these mechanisms are crucial to curtail the obesity epidemic.

## Supporting Information

S1 Fig Food and water intake normalized to fat free mass (FFM). Panel A) When normalized to g FFM, mice tended to eat more during week 15 compared to week 4 (*p* = 0.008), though there were no significant differences between groups (*p* = 0.17, repeated measures ANOVA). Panel B) When normalized to FFM, mice imbibed a similar quantity of water at week 4 compared to week 15 (p = 0.22). There were also no significant differences between groups (*p* = 0.80). *n* = 16 for those on the low glycemic response diet and *n* = 15 for those on the high glycemic response diet; repeated measures ANOVA. Data represent avg ± SE.
(DOCX)

S2 Fig Total activity counts per min averaged over all mice. Total activity counts per min were averaged over 16 mice in the low glycemic response group (blue) and 16 mice in the high glycemic response group (red) at week 4 (panel A) and week 15 (panel B). Lights were off between 19:00 and 7:00 (night/dark cycle).
(DOCX)

S3 Fig MESA analysis to elucidate ultradian rhythms. Data were pooled into 10 min bins. The effects of day/night were removed with a 4 h, 2-pole Butterworth high pass filter before being subjected to maximum entropy spectral analysis (MESA) analysis (Dowse, 2013). The spectral density indicates the strength of the oscillation at the given time period.
(DOCX)

S4 Fig Metabolic acclimation to a high fat diet. During this phase of the experiment, the mice maintained the HGR (gray) or LGR (white) starches in their diets. Panel A) Body mass increase upon subjection to a high fat (42.7% kcal) for 4 weeks. (*p* = 0.11 when comparing rate of weight gain between groups). Panel B) Resting energy expenditure (RER) during the day and at night during week 20 (the fourth week on the high fat diet). Panel C) Heat production (i.e, energy expenditure) during week 20. *n* = 10 in the HGR group and *n* = 9 in the LGR group. Data represent avg ± SE.
(DOCX)

S1 Table Ingredient composition of the experimental diets. (DOCX)

S2 Table Correlation coefficients between statistically significant measures. (DOCX)
