# Supplementary material for: The Intra- or Extracellular Redox State Was Not Affected by a High vs. Low Glycemic Response Diet in Mice
Source: PLoS One. 2015 Jun 1;10(6):e0128380. doi: 10.1371/journal.pone.0128380 (PMC4451145; doi:10.1371/journal.pone.0128380)
Supplement: S1 Fig — Panel A) When normalized to g FFM, mice tended to eat more during week 15 compared to week 4 (p = 0.008), though there were no significant differences between groups (p = 0.17, repeated measures ANOVA). Panel B) When normalized to FFM, mice imbibed a similar quantity of water at week 4 compared to week 15 (p = 0.22). There were also no significant differences between groups (p = 0.80). n = 16 for those on the low glycemic response diet and n = 15 for those on the high glycemic response diet; repeated measures ANOVA. Data represent avg ± SE. (DOCX) [file pone.0128380.s001.docx]

## Kleckner et al.

## A high or low glycemic response diet does not affect the intra- or extracellular redox state in mice

## Supporting Material


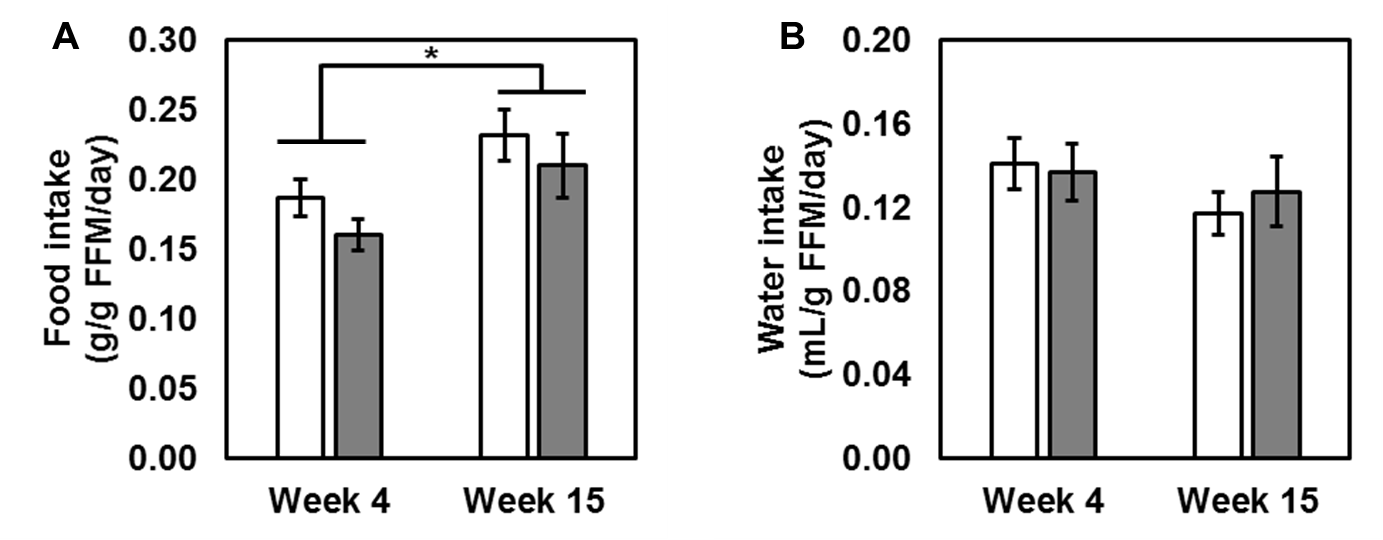


**Supporting Figure S1.** **Food and water intake normalized to fat free mass (FFM)** A) When normalized to g FFM, mice tended to eat more during week 15 compared to week 4 (*p* = 0.008), though there were no significant differences between groups (*p* = 0.17, repeated measures ANOVA). B) When normalized to FFM, mice imbibed a similar quantity of water at week 4 compared to week 15 (p = 0.22). There were also no significant differences between groups (*p* = 0.80). *n* = 16 for those on the low glycemic response diet and *n* = 15 for those on the high glycemic response diet; repeated measures ANOVA. Data represent avg ± SE.
