# Supplementary material for: The Intra- or Extracellular Redox State Was Not Affected by a High vs. Low Glycemic Response Diet in Mice
Source: PLoS One. 2015 Jun 1;10(6):e0128380. doi: 10.1371/journal.pone.0128380 (PMC4451145; doi:10.1371/journal.pone.0128380)
Supplement: S2 Fig — Total activity counts per min were averaged over 16 mice in the low glycemic response group (blue) and 16 mice in the high glycemic response group (red) at week 4 (panel A) and week 15 (panel B). Lights were off between 19:00 and 7:00 (night/dark cycle). (DOCX) [file pone.0128380.s002.docx]

## Kleckner et al.

## A high or low glycemic response diet does not affect the intra- or extracellular redox state in mice

## Supporting Material


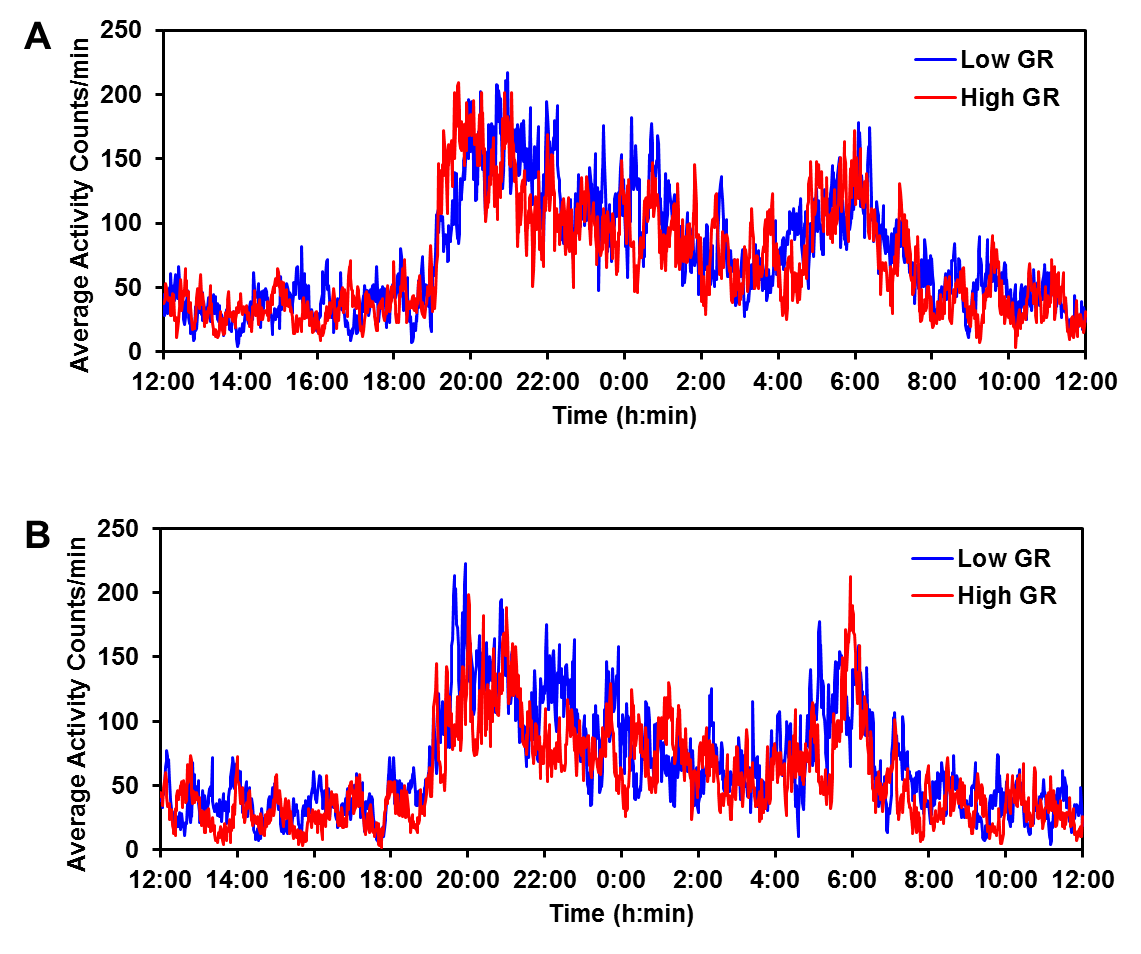


**Supporting Figure S2**. **Total activity counts per min averaged over all mice.** Total activity counts per min were averaged over 16 mice in the low glycemic response group (blue) and 16 mice in the high glycemic response group (red) at week 4 (panel A) and week 15 (panel B). Lights were off between 19:00 and 7:00 (night/dark cycle).
