# Supplementary material for: The Intra- or Extracellular Redox State Was Not Affected by a High vs. Low Glycemic Response Diet in Mice
Source: PLoS One. 2015 Jun 1;10(6):e0128380. doi: 10.1371/journal.pone.0128380 (PMC4451145; doi:10.1371/journal.pone.0128380)
Supplement: S3 Fig — Data were pooled into 10 min bins. The effects of day/night were removed with a 4 h, 2-pole Butterworth high pass filter before being subjected to maximum entropy spectral analysis (MESA) analysis (Dowse, 2013). The spectral density indicates the strength of the oscillation at the given time period. (DOCX) [file pone.0128380.s003.docx]

## Kleckner et al.

## A high or low glycemic response diet does not affect the intra- or extracellular redox state in mice

## Supporting Material


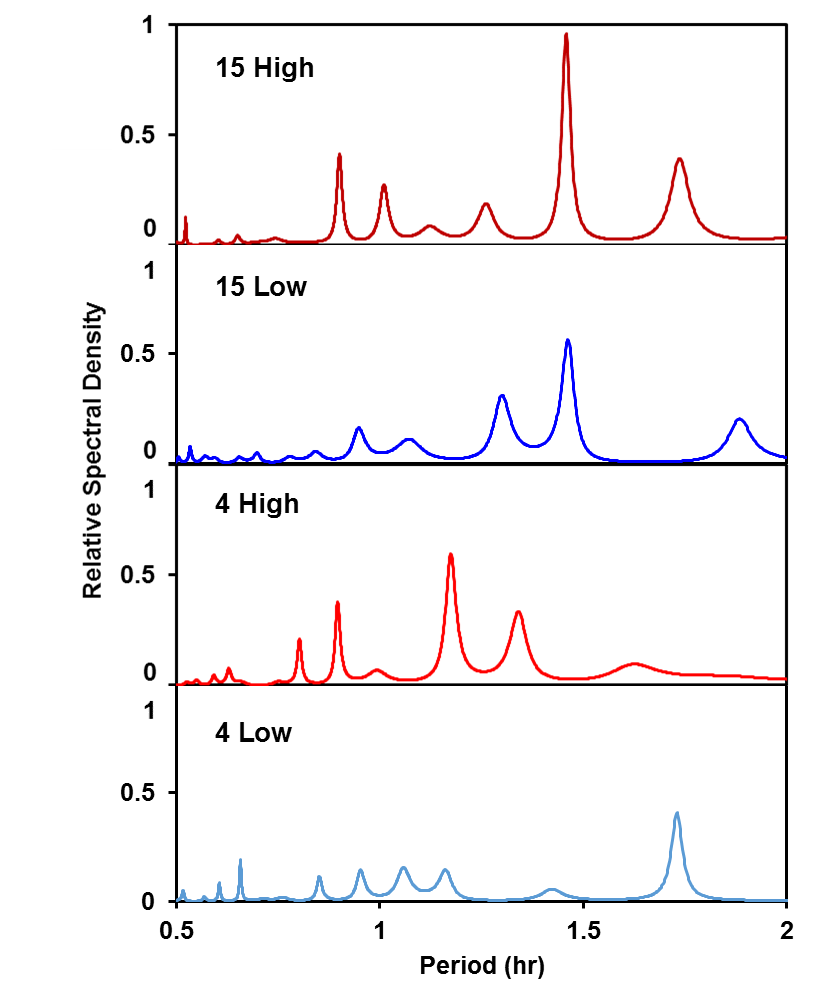


**Supporting Figure S3**. **MESA analysis to elucidate ultradian rhythms.** Data were pooled into 10 min bins. The effects of day/night were removed with a 4 h, 2-pole Butterworth high pass filter before being subjected to maximum entropy spectral analysis (MESA) analysis (Dowse, 2013). The spectral density indicates the strength of the oscillation at the given time period.
