# Supplementary material for: The Intra- or Extracellular Redox State Was Not Affected by a High vs. Low Glycemic Response Diet in Mice
Source: PLoS One. 2015 Jun 1;10(6):e0128380. doi: 10.1371/journal.pone.0128380 (PMC4451145; doi:10.1371/journal.pone.0128380)
Supplement: S4 Fig — During this phase of the experiment, the mice maintained the HGR (gray) or LGR (white) starches in their diets. Panel A) Body mass increase upon subjection to a high fat (42.7% kcal) for 4 weeks. (p = 0.11 when comparing rate of weight gain between groups). Panel B) Resting energy expenditure (RER) during the day and at night during week 20 (the fourth week on the high fat diet). Panel C) Heat production (i.e, energy expenditure) during week 20. n = 10 in the HGR group and n = 9 in the LGR group. Data represent avg ± SE. (DOCX) [file pone.0128380.s004.docx]

## Kleckner et al.

## A high or low glycemic response diet does not affect the intra- or extracellular redox state in mice

## Supporting Material


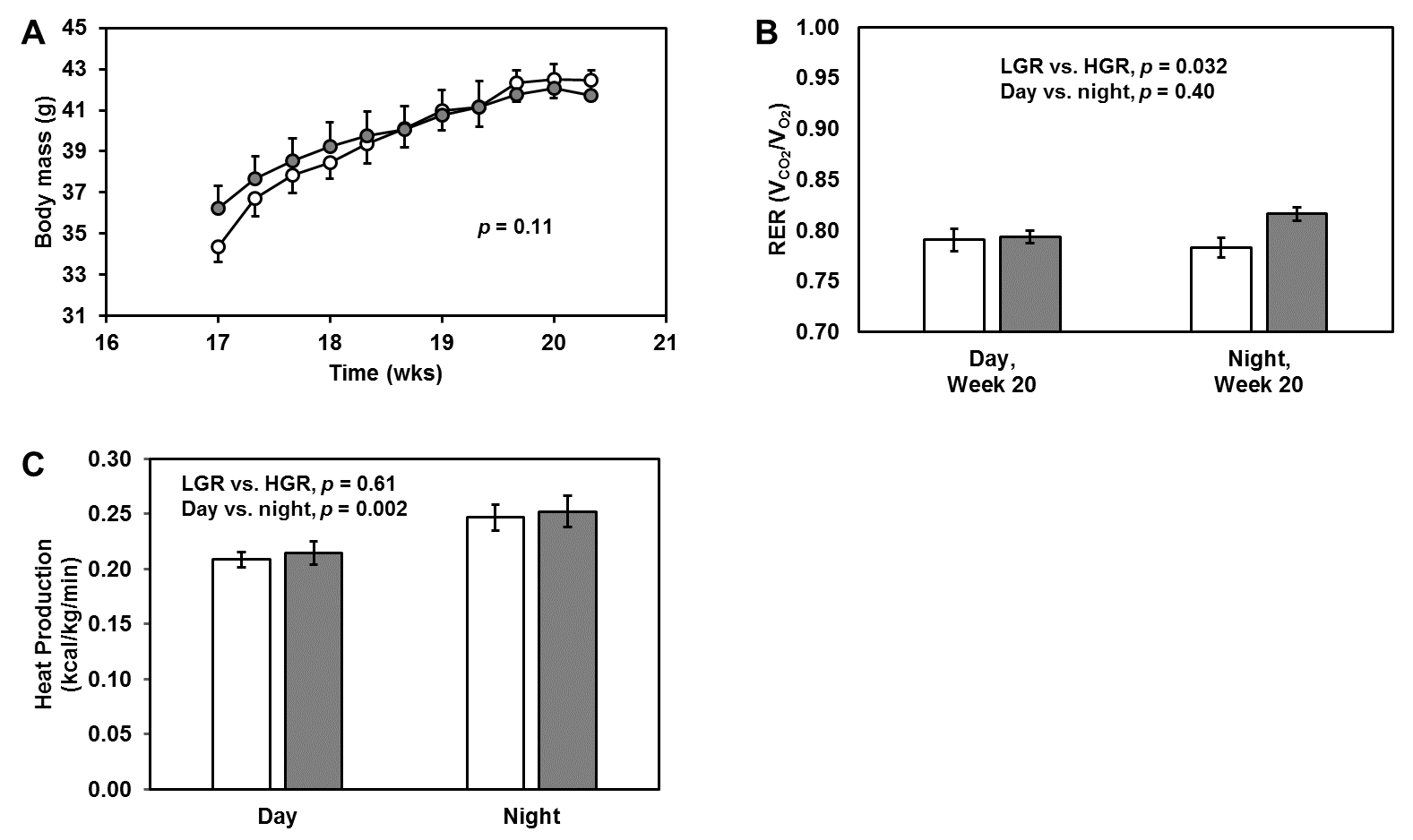


**Supplemental Figure 4**. **Metabolic acclimation to a high fat diet.** During this phase of the experiment, the mice maintained the HGR (gray) or LGR (white) starches in their diets. A) Body mass increase upon subjection to a high fat (42.7% kcal) for 4 weeks. (*p* = 0.11 when comparing rate of weight gain between groups). B) Resting energy expenditure (RER) during the day and at night during week 20 (the fourth week on the high fat diet). C) Heat production (i.e, energy expenditure) during week 20. *n* = 10 in the HGR group and *n* = 9 in the LGR group. Data represent avg ± SE.
