# Supplementary material for: The Intra- or Extracellular Redox State Was Not Affected by a High vs. Low Glycemic Response Diet in Mice
Source: PLoS One. 2015 Jun 1;10(6):e0128380. doi: 10.1371/journal.pone.0128380 (PMC4451145; doi:10.1371/journal.pone.0128380)
Supplement: S1 Table — (DOCX) [file pone.0128380.s005.docx]

## Kleckner et al.

## A high or low glycemic response diet does not affect the intra- or extracellular redox state in mice

## Supporting Material

## Supporting Table S1. Ingredient composition of the experimental diets

|  | **Low GR** | | **High GR** | | **High Fat, Low GR** | | **High fat, High GR** | |
| --- | --- | --- | --- | --- | --- | --- | --- | --- |
| **Ingredient** | **g %** | **kcal %** | **g %** | **kcal%** | **g %** | **kcal %** | **g %** | **kcal %** |
| Protein | 20.3 | 20.1 | 20.3 | 20.1 | 23.1 | 20.1 | 23.1 | 20.1 |
| Carbohydrate | 67.0 | 66.5 | 67.0 | 66.5 | 51.4 | 44.8 | 51.4 | 44.8 |
| Fat | 6.0 | 13.4 | 6.0 | 13.4 | 17.9 | 35.1 | 17.9 | 35.1 |
| Total | 93.3 | 100.0 | 93.3 | 100.0 | 92.4 | 100.0 | 92.4 | 100.0 |
| kcal/g | 4.03 |  | 4.03 |  | 4.59 |  | 4.59 |  |
|  |  |  |  |  |  |  |  |  |
| **Ingredient** |  |  |  |  |  |  |  |  |
| Casein | 200 | 800 | 200 | 800 | 200 | 800 | 200 | 800 |
| dl-methionine | 3 | 12 | 3 | 12 | 3 | 12 | 3 | 12 |
| High amylose starch | 550 | 2200 | 0 | 0 | 331.6 | 1326.5 | 0 | 0 |
| Waxy maize starch | 0 | 0 | 550 | 2200 | 0 | 0 | 331.6 | 1326.5 |
| Sucrose | 0 | 0 | 0 | 0 | 0 | 0 | 0 | 0 |
| Maltodextrin | 110 | 440 | 110 | 440 | 110 | 440 | 110 | 440 |
| Cellulose | 30 | 0 | 30 | 0 | 30 | 0 | 30 | 0 |
| Soybean oil | 60 | 540 | 60 | 540 | 60 | 540 | 60 | 540 |
| Primex (shortening) | 0 | 0 | 0 | 0 | 97.0 | 873.3 | 97.0 | 873.3 |
| Mineral mix*^a^* | 35 | 0 | 35 | 0 | 35 | 0 | 35 | 0 |
| Vitamin mix*^b^* | 10 | 40 | 10 | 40 | 10 | 40 | 10 | 40 |
| Choline bitartrate | 2 | 0 | 2 | 0 | 2 | 0 | 2 | 0 |
| FD&C yellow dye #5 | 0.025 | 0 | 0 | 0 | 0.015 | 0 | 0 | 0 |
| FD&C red dye #40 | 0 | 0 | 0.05 | 0 | 0 | 0 | 0.030 | 0 |
| FD&C blue dye #1 | 0.025 | 0 | 0 | 0 | 0.035 | 0 | 0.020 | 0 |
| Total | 1000 | 4032 | 1000 | 4032 | 878.7 | 4031.8 | 878.7 | 4031.8 |

*^a^*AIN-76A, S10001, Research Diets, Inc.

*^b^*AIN-76A, V10001, Research Diets, Inc.
