# Supplementary material for: The Intra- or Extracellular Redox State Was Not Affected by a High vs. Low Glycemic Response Diet in Mice
Source: PLoS One. 2015 Jun 1;10(6):e0128380. doi: 10.1371/journal.pone.0128380 (PMC4451145; doi:10.1371/journal.pone.0128380)
Supplement: S2 Table — (DOCX) [file pone.0128380.s006.docx]

## Kleckner et al.

## A high or low glycemic response diet does not affect the intra- or extracellular redox state in mice

## Supporting Material

## Supporting Table S2. Correlation coefficients between statistically significant measures*^a^*

| P1*^b^* | P2*^b,c^* | r*^d^* | *p*-value | Corrected *p*-value*^e^* |
| --- | --- | --- | --- | --- |
| StartingMass | Heat4 | -0.675 | 1.61E-05 | 1.53E-02 |
| StartingMass | TotGSHL | -0.744 | 6.80E-07 | 6.43E-04 |
| RateGain | Fat12 | 0.885 | 7.77E-12 | 7.35E-09 |
| RateGain | Lean12 | -0.865 | 8.56E-11 | 8.10E-08 |
| RateGain | TotWater12 | -0.842 | 8.04E-10 | 7.61E-07 |
| RateGain | Fat15 | 0.871 | 4.19E-11 | 3.96E-08 |
| RateGain | Lean15 | -0.851 | 3.41E-10 | 3.23E-07 |
| RateGain | TotWater15 | -0.822 | 4.44E-09 | 4.20E-06 |
| Heat4 | TotGSHL | 0.664 | 2.54E-05 | 2.40E-02 |
| Fat4 | Lean4 | -0.775 | 1.18E-07 | 1.11E-04 |
| Fat4 | TotWater4 | -0.778 | 1.02E-07 | 9.64E-05 |
| Fat4 | Fat12 | 0.757 | 3.42E-07 | 3.23E-04 |
| Fat4 | Lean12 | -0.777 | 1.06E-07 | 1.00E-04 |
| Fat4 | TotWater12 | -0.763 | 2.38E-07 | 2.25E-04 |
| Fat4 | Fat15 | 0.713 | 3.17E-06 | 2.99E-03 |
| Fat4 | Lean15 | -0.712 | 3.40E-06 | 3.22E-03 |
| Fat4 | TotWater15 | -0.687 | 1.00E-05 | 9.46E-03 |
| Lean4 | TotWater4 | 0.790 | 4.73E-08 | 4.47E-05 |
| Fat12 | Lean12 | -0.992 | 2.98E-29 | 2.81E-26 |
| Fat12 | TotWater12 | -0.984 | 9.28E-25 | 8.78E-22 |
| Fat12 | Fat15 | 0.962 | 4.20E-19 | 3.97E-16 |
| Fat12 | Lean15 | -0.935 | 1.59E-15 | 1.50E-12 |
| Fat12 | TotWater15 | -0.927 | 8.65E-15 | 8.19E-12 |
| Fat12 | SubQ | 0.698 | 6.31E-06 | 5.97E-03 |
| Lean12 | TotWater12 | 0.988 | 8.47E-27 | 8.01E-24 |
| Lean12 | Fat15 | -0.962 | 5.65E-19 | 5.34E-16 |
| Lean12 | Lean15 | 0.949 | 4.57E-17 | 4.32E-14 |
| Lean12 | TotWater15 | 0.934 | 2.24E-15 | 2.12E-12 |
| Lean12 | SubQ | -0.693 | 7.85E-06 | 7.43E-03 |
| TotWater12 | Fat15 | -0.962 | 4.28E-19 | 4.05E-16 |
| TotWater12 | Lean15 | 0.948 | 5.04E-17 | 4.77E-14 |
| TotWater12 | TotWater15 | 0.953 | 1.20E-17 | 1.13E-14 |
| TotWater12 | SubQ | -0.712 | 3.33E-06 | 3.15E-03 |
| Food15 | AvgTemp | -0.700 | 5.79E-06 | 5.48E-03 |
| Fat15 | Lean15 | -0.978 | 1.26E-22 | 1.19E-19 |
| Fat15 | TotWater15 | -0.972 | 4.99E-21 | 4.72E-18 |
| Fat15 | SubQ | 0.712 | 3.31E-06 | 3.13E-03 |
| Lean15 | TotWater15 | 0.986 | 8.14E-26 | 7.70E-23 |
| Lean15 | SubQ | -0.685 | 1.08E-05 | 1.02E-02 |
| TotWater15 | SubQ | -0.692 | 8.21E-06 | 7.76E-03 |
| SubQ | Epi | 0.772 | 1.42E-07 | 1.34E-04 |
| Pyruvate | AvgTemp | -0.651 | 4.11E-05 | 3.88E-02 |
| B:A | TBARSliver | -0.672 | 1.87E-05 | 1.77E-02 |
| TBARSliver | TotGSHB | -0.711 | 3.61E-06 | 3.42E-03 |
| TBARSliver | TotGSHL | -0.785 | 6.16E-08 | 5.83E-05 |
| TotGSHB | TotGSHL | 0.656 | 3.41E-05 | 3.23E-02 |

*^a^*The Bonferroni correction was applied conservatively so that *p*-values of less than 5.29e-5 were deemed significant.

*^b^*P1 and P2 = parameters 1 and 2 (arbitrarily assigned).

*^c^*See Fig. 6 caption for explanation of parameter abbreviations

*^d^*Negative correlations are in red.

*^e^*The uncorrected *p*-value × 946 to yield a conservative probability that these results would be obtained due to chance
